# Umbilical Cord Blood Gas Analysis, Obstetric Performance and Perinatal Outcome

**DOI:** 10.1055/s-0038-1675187

**Published:** 2018-10-30

**Authors:** Cátia Sofia Ferreira, Ângela Melo, Ana Helena Fachada, Helena Solheiro, Nuno Nogueira Martins

**Affiliations:** 1 Department of Obstetrics and Gynecology, Centro Hospitalar Tondela-Viseu, Viseu, Portugal

**Keywords:** Umbilical Cord Blood Gas Analysis, delivery, morbidity, neonatology, gasometria do cordão umbilical, parto, morbilidade, neonatologia

## Abstract

**Objective** To analyze if umbilical artery pH (pH_ua_) ≤7.00 and umbilical artery blood deficit (BD_ua_) ≥12.00 mmol/L are good predictors of adverse neonatal outcomes.

**Methods** This was an observational, longitudinal and retrospective cohort study, conducted at the department of obstetrics and gynecology of Centro Hospitalar Tondela Viseu between September 2013 and September 2015. Total cohort and subgroup analysis were performed: group A—women with umbilical cord blood gas analysis (UCBGA) performed for non-reassuring fetal cardiotocographic patterns, placental abruption, or shoulder dystocia; and group B—all the others. Assays were made with the software SPSS for Windows, Versions 20.0 and 21.0 (IBM Corp., Armonk, NY, USA).

**Results** A total of 428 UCBGAs met the inclusion criteria. The group analysis revealed an association between group A and pHua ≤7.00, as well as between BDua ≥12.00 mmol/L and 1st minute Apgar score ≤4 (*p* = 0.011). After the application of the logistic regression models in the total cohort analysis, pH_ua _≤7.00 had an impact in the occurrence of acute neonatal hypoxia (odds ratio [OR]: 6.71; 95% confidence interval [CI]: 1.21–37.06; *p* = 0.029); multiparous women had a higher risk of delivering a newborn with first minute Apgar score ≤4 and acute neonatal hypoxia (OR: 5.38; 95% CI: 1.35–21.43; *p* = 0.017; and OR: 2.66; 95% CI: 1.03–6.89, *p* = 0.043, respectively); women who had urologic problems during pregnancy had a higher risk of delivering a newborn with 5th minute Apgar score ≤7 (OR: 15.17; 95% CI: 1.29–177.99; *p* = 0.030); and shoulder dystocia represented a 15 times higher risk of acute neonatal hypoxia (OR: 14.82; 95% CI: 2.20–99.60; *p* = 0.006).

**Conclusion** The pH_ua_ and the BD_ua_ are predictors of adverse neonatal outcome, and UCBGA is a useful tool for screening newborns at risk. Universal UCBGA should be considered for all deliveries, as it is an accurate screening test for neonatal hypoxia.

## Abstract (pt)

**Resumo:**

**Objetivo** Avaliar se o pH da artéria umbilical (pH_ua_) ≤7,00 e o déficit de bases da artéria umbilical (BD_ua_) ≥12,00 mmol/L são preditores de desfechos neonatais adversos.

**Métodos** Estudo observacional, longitudinal e retrospectivo, realizado no Serviço de Ginecolocgia e Obstetrícia do Centro Hospitalar Tondela Viseu durante o período de setembro de 2013 a setembro de 2015. Foi realizada a análise de toda a coorte e de dois subgrupos: grupo A—mulheres cuja gasometria do cordão umbilical (UCBGA, na sigla em inglês) foi realizada por traçado cardiotocográfico não tranquilizador, descolamento prematuro de placenta normalmente inserida, ou distócia de ombros; e grupo B—todas as outras. A análise estatística foi realizada com o programa SPSS for Windows, Versões 20.0 e 21.0 (IBM Corp., Armonk, NY, USA)

**Resultados** Um total de 428 UCBGAs cumpriram os critérios de inclusão. A análise de grupo revelou uma associação entre o grupo A e pH_ua _≤7,00 (*p* = 0,002), e entre BD_ua _≥12,00 mmol/L e índice de Apgar ao 1^o^ minuto ≤4 (*p* = 0,011). Após a aplicação dos modelos de regressão logística na análise da coorte total, pH_ua _≤7.00 teve impacto na ocorrência de hipóxia neonatal aguda (razão de probabilidade [RP]: 6,71; 95% índice de confiança [IC]: 1,21–37,06; *p* = 0,029); verificou-se maior risco de recém-nascido com índice de Apgar ao 1^o^ minuto ≤4 e hipóxia neonatal aguda nas multíparas (RP: 5,38; 95% IC: 1,35–21,.43; *p* = 0,017; e RP: 2,66; 95% IC: 1,03–6,89; *p* = 0,043, respectivamente); e de recém-nascido com índice de Apgar ao quinto minuto ≤7 nas mulheres com problemas urológicos na gravidez (RP: 15,17; 95% IC: 1,29–177,99; *p* = 0,030); e a ocorrência de distócia de ombros aumentou 15 vezes o risco de hipóxia neonatal aguda (RP: 14,82; 95% IC: 2,20–99,60; *p* = 0,006).

**Conclusão** O pH_ua_ e o BD_ua_ são preditores de desfecho neonatal adverso, e a UCBGA é uma ferramenta útil no rastreio dos recém-nascidos em risco. A realização universal de UCBGA deve ser considerada em todos os partos, visto ser um teste de rastreio objetivo de hipóxia neonatal.

## Introduction

Fetal and neonatal acidemia are associated with several adverse neonatal outcomes.^1^ ^2^ ^3^ ^4^ ^5^ ^6^ ^7^ These outcomes include low Apgar scores, respiratory distress syndrome (RDS), hypoxic-ischemic encephalopathy (HIE), seizures, intraventricular hemorrhage, sepsis, and death.^1^ ^2^ ^3^ ^4^ ^5^ ^6^ ^7^ Determining the time of injury is essential to understand the mechanisms underlying these outcomes and can have important medico-legal implications. The umbilical cord blood gas analysis (UCBGA) is an objective and validated tool to evaluate the oxygenation and metabolic status of the newborn at birth.^1^ ^2^ ^3^ ^4^ ^5^ ^6^ If the umbilical artery pH (pH_ua_) is ≤7.00 and the umbilical artery base deficit (BD_ua_) is ≥12.00 mmol/L, there is an established diagnosis of neonatal metabolic acidemia, and the risk of neurologic sequelae is higher in this setting.^1^ ^2^ ^3^ ^6^ ^8^ Although the pH_ua_ is a well-established marker of neonatal hypoxia, the BD_ua_ is not. The BD_ua_ is useful in distinguishing between respiratory and metabolic umbilical artery acidemia.^1^ ^2^ ^3^ ^4^ ^5^ ^6^ ^9^ This distinction is important because respiratory acidosis is not usually associated with complications for the newborn.^10^ ^11^ Performing an UCBGA remains a good screening test for newborns at risk of poor neurologic outcome.^4^ ^12^ ^13^ It can be recommended for all high-risk deliveries.^4^ ^12^ The Royal College of Obstetricians and Gynaecologists recommends that an UCBGA should be performed in all cesarean or operative vaginal deliveries executed due to fetal compromise.^12^ The American College of Obstetricians and Gynecologists states that an UCBGA should be performed after any delivery in which a fetal metabolic abnormality is suspected.^13^ The main aim of the present study was to analyze whether pH_ua _≤7.00 and BD_ua _≥12.00 mmol/L were good predictors of adverse neonatal outcomes. The secondary aim of the present study was to determine if there was any association between these outcomes and other potential risk factors that could act as confounders.

## Methods

This was an observational, longitudinal and retrospective cohort study conducted between September 2013 and September 2015, at the department of obstetrics and gynecology of Centro Hospitalar Tondela Viseu. All women who delivered in the labor ward and had a valid UCBGA according to the local protocol (Table 1) were included.

**Table 1:** Indications for performing umbilical cord blood gas analysis

Maternal thyroid disease
Assisted reproductive technology
Multiple pregnancy
Fetal growth restriction and/or another fetal pathology
Non-reassuring fetal cardiotocographic pattern
Intrapartum fever
Preterm delivery
Instrumental delivery
Low Apgar score (< 8 at any minute) or decreasing Apgar score
Low birthweight (< 2,500 g) or small newborn for gestational age

**Fig. 1:**
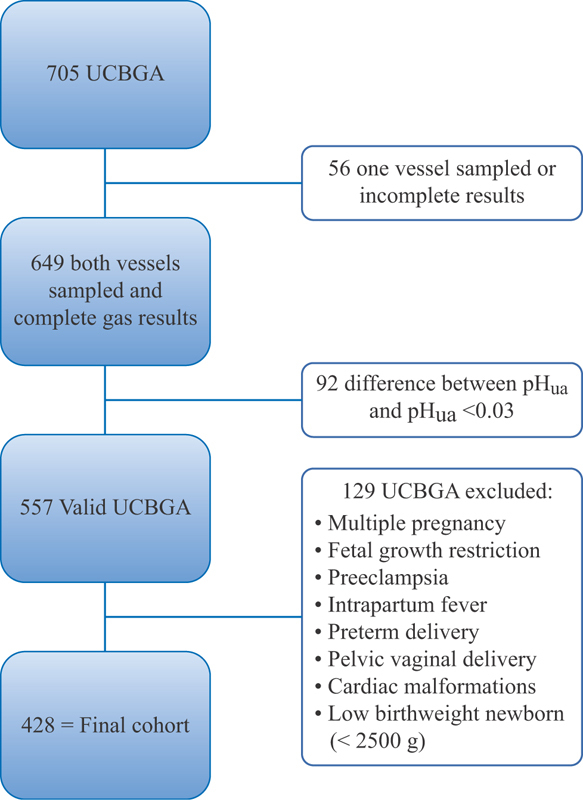
Study's sample exclusion criteria.

All cases of multiple pregnancies, fetal growth restrictions, preeclampsia, intrapartum fever, preterm delivery, pelvic vaginal delivery, antenatal or postnatal diagnosis of cardiac malformations and low birthweight newborn were excluded (Fig. 1). Valid samples had a difference of ≥0.03 between pH_ua_ and umbilical vein pH.

The total cohort data was analyzed. A subsequent group analysis was also done: group A, which included the UCBGA performed for non-reassuring fetal cardiotocography (CTG) patterns or placental abruption, and group B, which included all the remaining cases. The purpose of the group analysis was to check for a stronger association of pH_ua_ ≤7.00 and BDua ≥12.00 mmol/L in group A and to find out if the predictive value of pH_ua_ ≤7.00 and BDua ≥12.00 mmol/L for adverse neonatal outcomes was stronger in group A than in group B. Data was collected through the review of electronic and manuscript clinical files.

In the UCBGA technique used, the cord was triple-clamped immediately after delivery with an in-between length of ∼ 10 to 20 cm. The distal segment was then cut and placed over a table, where the blood samples were taken, first from the artery and then from the vein. Preheparinised syringes (Pro-Vent^®^, Smiths medical ASD, Inc., Keene, New Hampshire, USA) were used. The samples were analyzed within a maximum of 30 minutes at the hospital central laboratory, and the results were made electronically available to the team immediately after.

Two neonatal outcome predictors were analyzed: pH_ua_ ≤7.00 and BD_ua _≥12.00 mmol/L.

The adverse neonatal outcomes that were analyzed were:

1. First minute Apgar Score (1′AS) ≤4;
2. Fifth minute Apgar Score (5′AS)  ≤ 7;
3. Acute neonatal hypoxia (ANH)—condition in which the newborn has been exposed to intrapartum reversible asphyxia, without documented HIE;
4. Hypoxic-ischemic encephalopathy—disturbed neurologic function in the earliest days of life, manifested by a reduced level of consciousness or seizures, often accompanied by difficulty at initiating and maintaining respiration, and by depression of tone and reflexes, in a newborn with one or more of the following: (a) a sentinel hypoxic or ischemic event occurring immediately before or during labor and delivery; (b) pH_ua_ ≤7.00 and/or BD_ua _≥12.00 mmol/L; (c) presence of multisystem organ failure consistent with HIE;
5. Neonatal convulsion;
6. Hyaline membrane disease or respiratory distress syndrome type I (RDS I);
7. Transient tachypnea of the newborn or respiratory distress syndrome type II (RDS II);
8. Meconium aspiration syndrome—respiratory distress at or shortly after birth in the presence of evidence of meconium-stained amniotic fluid on infant and characteristic radiographic features (hyperinflation of the lungs with flattening of the diaphragm, diffuse patchy densities alternating with areas of expansion, pneumothorax, and pneumomediastinum);
9. Neonatal bradycardia;
10. Neonatal sepsis—a clinically ill newborn with isolation of pathogenic bacteria from blood culture or, in its absence, with laboratory test results consistent with infection (leukopenia or leukocytosis, immature to total neutrophil ratio > 0.16; immature to mature neutrophil ratio > 0.2; C-reactive protein > 10–15 mg/L and/or procalcitonin > 3 ng/mL); and after exclusion of other infectious causes.

The Analysis was performed using the software SPSS for Windows, Versions 20.0 and 21.0 (IBM Corp., Armonk, NY, USA). Maternal and newborn demographic data and the incidence of outcomes were compared between the groups with the chi-squared test and the Fisher exact test. The association between each outcome and the predictors was made through univariate analysis, calculating the odds ratio (OR) with a 95% confidence interval (CI). The Wald test was applied to the outcomes with a statistically significantly association with one or both predictors to check if the ORs were different between the groups and to decide whether to analyze them separately for each group or in the total cohort. Independent predictors of three outcomes were obtained with two multivariate analyses with multiple logistic regressions. The multivariate analysis models were tested with the Hosmer and Lemeshow test and receiver operating characteristic curves. The possible confounders tested were: (a) maternal age (< 35 versus ≥35 years old); (b) parity (nulliparity versus multiparity); (c) gestational age (37–38 versus ≥39 weeks); (d) presence of insulin treated and non-insulin treated gestational diabetes; (e) urologic problems during pregnancy (such as recurrent cystitis, pyelonephritis, hydronephrosis, and hydroureter); (e) presence of oligohydramnios; (f) mode of delivery; (g) shoulder dystocia at birth; (h) newborn gender; (i) newborn birthweight (< 4,000 g versus ≥4,000 g); (j) presence of neonatal sepsis; and (k) presence of neonatal seizures. The significance level was 5% (*p *< 0.05).

The Ethics Committee for Health of the authors' institution approved the present study under the reference number 21/4/2017/1, on April 21st, 2017.

## Results

The main characteristics of the cohort are listed in Table 2.

**Table 2:** Characteristics of the cohort

Variables	Total cohort		Group A		Group B		
	*n*	%	*n*	%	*n*	%	*p*-value
Maternal age (years old)							0.957
≤ 19	10	2.3	1	0.6	9	3.6	
20–24	36	8.4	16	9.1	20	7.9	
25–29	117	27.3	52	29.7	65	25.7	
30–34	158	36.9	62	35.4	96	37.9	
35–37	57	13.3	27	15.4	30	11.9	
≥ 38	50	11.7	17	9.7	33	13.0	
Parity							0.092
Nulliparous	291	68.0	111	63.4	180	71.1	
Multiparous	137	21.0	64	36.6	73	28.9	
Insulin treated gestational diabetes							0.714
No	409	95.6	168	96.0	241	95.3	
Yes	19	4.4	7	4.0	12	4.7	
Non-insulin treated gestational diabetes							1.000
No	419	97.9	171	97.7	248	98.0	
Yes	9	2.1	4	2.3	5	2.0	
Gestational hypertension							0.481
No	420	98.1	173	97.7	247	97.6	
Yes	8	1.9	2	2.3	6	2.4	
Urologic problems during pregnancy							0.164
No	423	98.8	171	97.7	252	99.6	
Yes	5	1.2	4	2.3	1	0.4	
Oligohydramnios							0.481
No	420	98.1	173	98.9	247	97.6	
Yes	8	1.9	2	1.1	6	2.4	
Gestational age at birth (gestational weeks)							0.667
37–38	75	17.5	29	16.6	46	18.2	
≥ 39	353	82.5	146	83.4	207	81.8	
Delivery							0.000
Normal vaginal	25	5.8	9	5.1	16	6.3	
Vacuum	239	55.8	80	55.8	159	62.8	
Forceps	30	7.0	9	5.1	21	8.3	
Caesarean section	134	31.3	175	44.0	57	22.5	
UCBGA motive							0.000
Control	14	3.3	0	0.0	14	5.5	
Assisted reproductive technology	1	0.2	0	0.0	1	0.4	
Small for gestational age newborn	1	0.2	0	0.0	1	0.4	
Instrumental delivery	235	54.9	0	0.0	235	92.9	
Non-reassuring fetal CTG pattern	171	40.0	171	97.7	0	0.0	
Placental abruption	4	0.9	4	2.3	0	0.0	
Shoulder dystocia	2	0.5	0	0.0	2	0.8	
Gender							0.015
Female	184	43.0	63	36.0	121	47.8	
Male	244	57.0	112	64.0	132	52.2	
Birthweight (grams)							0.013
< 4,000	415	97.0	174	99.4	241	95.3	
≥ 4,000	13	3.0	1	0.6	12	4.7	
Total	428	100.0	175	100.0	253	100.0	

Abbreviations: CTG, cardiotocographic; UCBGA, umbilical cord blood gas analysis.

The mean maternal age did not differ significantly between the groups; it was 31.0 ± 5.1 and 30.8 ± 5.3 years old in groups A and B, respectively (*p* = 0.809). In addition, there were no major differences in the distribution of maternal age between the age groups (*p* = 0.957). In both groups, the majority of women was nulliparous (63.4% in group A versus 71.1% in group B, *p* = 0.092). Few women had a diagnosis of gestational diabetes, gestational hypertension, or urologic problems during pregnancy. Only two women in group A and six women in group B had oligohydramnios (*p* = 0.481).

The mean gestational age at delivery was similar between the groups (39.45 ± 1.02 weeks in group A versus 39.36 ± 1.06 weeks in group B; *p* = 0.380), and most women delivered at 39 weeks or beyond (83.4% in group A versus 81.8% in group B; *p* = 0.667). Vacuum assisted delivery was the most common birth type in both groups (45.7% versus 62.8% in groups A and B, respectively) and the caesarean rate was higher in group A (44.4% versus 22.5%; *p* = 0.000). The most frequent reason to perform an UCBGA in group A was a non-reassuring fetal CTG pattern (*n* = 171; 97.7%), while in group B it was instrumental delivery (*n* = 235; 92.9%). The women in group A were more likely to have male newborns (64.0% versus 52.2%; *p* = 0.015), although the male gender predominated in both groups. The mean birthweight was higher in group B (3,254.14 ± 337.48 versus 3,345.85 ± 407.09 g; *p* = 0.012) and the women in group B were more likely to have macrosomic newborns (0.6% versus 4.7%; *p* = 0.013).

In group A, the mean pH_ua_ was lower (7.16 ± 0.10 versus 7.20 ± 0.08; *p* = 0.000) and the mean BD_ua_ was higher (5.10 ± 3.84 versus 4.12 ± 3.74 mmol/L; *p* = 0.009) than in group B. The newborns from group A were more likely to have pH_ua _≤7.00, but not BD_ua _≥12.00 mmol/L (*p* = 0.002 and *p* = 0.721, respectively) (Table 3).

**Table 3:** Descriptive statistics of neonatal outcome predictors

Variables	Total cohort		Group A		Group B		
	*n*	%	*n*	%	*n*	%	*p*-*value*
pH_ua_							0.002
≤ 7.00	17	4.0	13	7.4	4	1.6	
> 7.00	411	96.0	162	92.6	249	98.4	
BD_ua_ (mmol/L)							0.721
≥ 12.00	8	1.9	4	2.3	4	1.6	
< 12.00	420	98.1	171	97.7	249	98.4	

Abbreviations: BD_ua, _umbilical artery blood deficit; pH_ua, _umbilical artery pH.

All eight cases of BD_ua _≥12.00 mmol/L in group A also had pH_ua_ ≤7.00. Considering the total cohort, newborns with pH_ua _≤7.00 were more likely to have BD_ua _≥12.00 mmol/L (*p* = 0.000) and vice-versa (*p* = 0.000). There were no statistically significant differences between the groups pH_ua _≤7.00 and pH_ua _> 7.00 and the groups BD_ua _≥12.00 mmol/L and BD_ua _< 12.00 mmol/L regarding any of the potentially confounders analyzed. Among all newborns, 26 in group A and 23 in group B had at least one adverse neonatal outcome (14.9% versus 9.1%; *p* = 0.065) (Table 4).

**Table 4:** Descriptive statistics of neonatal outcomes

Variables	Total cohort		Group A		Group B		
	*n*	%	*n*	%	*n*	%	*p*-*value*
Adverse neonatal outcome							0.065
No	379	88.6	149	85.1	230	90.9	
Yes	49	11.4	26	14.9	23	9.1	
1′AS							0.078
≤ 4	416	97.2	8	4.6	4	1.6	
> 4	12	2.8	167	95.4	249	98.4	
5**′** AS							0.020
≤ 7	421	98.4	6	3.4	1	0.4	
> 7	7	1.6	169	96.6	252	99.6	
ANH							0.189
No	408	95.3	164	93.7	244	96.4	
Yes	20	4.7	11	6.3	9	3.6	
HIE							1.000
No	427	99.8	175	100.0	252	99.6	
Yes	1	0.2	0	0.0	1	0.4	
Neonatal seizures							1.000
No	426	99.5	174	99.4	252	99.6	
Yes	2	0.5	1	0.6	1	0.4	
RDS I							0.057
No	417	97.4	167	95.4	250	98.8	
Yes	11	2.6	8	4.6	3	1.2	
RDS II							0.203
No	412	96.3	9	5.1	246	97.2	
Yes	16	3.7	166	94.9	7	2.8	
Meconium aspiration syndrome							
No	424	99.1	173	98.9	251	99.2	1.000
Yes	4	0.9	2	1.1	2	0.8	
Neonatal bradycardia							
No	423	98.8	174	99.4	249	98.4	0.653
Yes	5	1.2	1	0.6	4	1.6	
Neonatal sepsis							1.000
No	424	99.1	173	98.9	251	99.2	
Yes	4	0.9	2	1.1	2	0.8	

Abbreviations: 1′ AS, first minute Apgar score; 5′ AS, fifth minute Apgar score; ANH, acute neonatal hypoxia; HIE, hypoxic-ischemic encephalopathy; RDS, respiratory distress syndrome.

The only neonatal outcome that had a statistically significant difference between the groups (3.4% versus 0.4%, *p* = 0.020) was 5′ AS ≤7. In the total cohort, there was only one case of HIE, which occurred in group B. It was a 40-week pregnancy, with the diagnosis of gestational hypertension and oligohydramnios, ending in a vacuum-assisted delivery due to a second stage arrest of labor. The 3,150 g male newborn had a 1′ AS = 3 and a 5′ AS = 7. The pH_ua_ was 6.85 and the BD_ua_ was 18.90. The results of the univariate regression analyses of association between neonatal outcomes and pH_ua _≤7.00 or BD_ua _≥12.00 mmol/L showed five significant associations (Table 5):

**Table 5:** Results of the univariate regression analyses for neonatal outcomes in newborns with pHua ≤7.00 and BDua ≥12.00 mmol/L

Variables	Total cohort			Group A			Group B		
	OR	95% CI	*p*-*value*	OR	95% CI	*p*-*value*	OR	95% CI	*p*-*value*
pH_ua _≤7.00									
1′AS ≤4	9.57	2.33–39.25	0.009	4.73	0.85–26.22	0.111	27.33	2.17–344.29	0.062
5′AS ≤7	10.83	1.94–60.40	0.028	2.62	0.28–24.23	0.375	n.a.	n.a.	0.016
ANH	7.60	2.23–25.91	0.006	3.09	0.59–16.09	0.191	34.57	4.24–282.06	0.007
Neonatal seizures	n.a.	n.a.	0.439	n.a.	n.a.	1.000	n.a.	n.a.	0.242
RDS I	2.51	0.30–20.79	0.363	1.85	0.21–16.26	0.468	n.a.	n.a.	1.000
RDS II	3.78	0.79–18.15	0.128	4.03	0.75–21.74	0.136	n.a.	n.a.	1.000
Meconium aspiration syndrome	n.a.	n.a.	1.000	n.a.	n.a.	1.000	n.a.	n.a.	1.000
Neonatal bradycardia	4.17	0.46–37.65	0.210	n.a.	n.a.	1.000	3.92	0.40–38.18	0.322
Neonatal sepsis	3.12	0.32–30.19	3.120	n.a.	n.a.	0.507	1.28	0.08–20.72	1.000
BD_ua _≥12.00 mmol/L									
1′AS ≤4	27.40	5.66–132.54	0.001	27.50	3.29–229.68	0.011	27.33	2.17–344.29	0.062
5′AS ≤7	9.86	1.04–93.05	0.125	n.a.	n.a.	0.016	n.a.	n.a.	1.000
ANH	7.44	1.40–39.49	0.049	5.37	0.51–56.36	0.230	10.04	0.94–107.45	0.136
Neonatal seizures	n.a.	n.a.	1.000	n.a.	n.a.	1.000	n.a.	n.a.	1.000
RDS I	n.a.	n.a.	1.000	n.a.	n.a.	1.000	n.a.	n.a.	1.000
RDS II	3.86	0.45–33–37	0.265	6.79	0.63–71.79	0.192	n.a.	n.a.	1.000
Meconium aspiration syndrome	n.a.	n.a.	1.000	n.a.	n.a.	1.000	n.a.	n.a.	1.000
Neonatal bradycardia	n.a.	n.a.	1.000	n.a.	n.a.	1.000	n.a.	n.a.	1.000
Neonatal sepsis	n.a.	n.a.	1.000	n.a.	n.a.	1.000	n.a.	n.a.	1.000

Abbreviations: 1′ AS, first minute Apgar score; 5′ AS, fifth minute Apgar score; ANH, acute neonatal hypoxia; BD_ua**,**_umbilical artery blood deficit; n.a., not applicable; pH_ua**,**_umbilical artery pH**.**; RDS, respiratory distress syndrome.

1. pH_ua _≤7.00 and 1′ AS ≤4 (total cohort: OR = 9.57; 95% CI: 2.33–39.25; *p* = 0.009);
2. pH_ua _≤7.00 and 5′ AS ≤7 (total cohort: OR = 10.83; 95% CI: 1.94–60.40; *p* = 0.028);
3. pH_ua _≤7.00 and ANH (total cohort: OR = 7.60; 95% CI: 2.23–25.91; *p* = 0.006; group B: OR = 34.57; 95% CI: 4.24–282.06; *p* = 0.007);
4. BD_ua _≥12.00 mmol/L and 1′ AS ≤4 (total cohort: OR = 27.40; 95% CI: 5.66–132.54; *p* = 0.001; group A: OR = 27.50; 95% CI: 3.29–229.68; *p* = 0.011);
5. BD_ua _≥12.00 mmol/L and ANH (total cohort: OR = 7.44; 95% CI: 1.40–39.49; *p* = 0.049).

In the total cohort, the univariate regression analysis of the association between these three neonatal outcomes and the possible confounding factors revealed that (Table 6):

**Table 6:** Results of the univariate regression analyses for confounding factors in newborns with 1′AS ≤4, 5′AS ≤7 and ANH, in total cohort

Variables	1′AS ≤ 4			5′AS ≤ 7			ANH		
	OR	95% CI	*p*-*value*	OR	95% CI	*p*-*value*	OR	95% CI	*p*-*value*
Study group	2.98	0.88–10.06	0.078	8.95	1.97–74.98	0.020	1.82	0.74–4.49	0.244
Maternal age	1.24	0.37–4.18	1.000	0.82	0.18–3.70	1.000	1.15	0.45–2.94	0.771
Parity	4.45	1.32–15.04	0.022	5.47	1.05–28.58	0.037	2.74	1.11–6.77	0.024
Insulin treated gestational diabetes	2.010	0.246	0.424	n.a.	n.a.	n.a.	n.a.	n.a.	n.a.
Non-insulin treated gestational diabetes	n.a.	n.a.	n.a.	n.a.	n.a.	n.a.	n.a.	n.a.	n.a.
Gestational hypertension	n.a.	n.a.	n.a.	n.a.	n.a.	n.a.	n.a.	n.a.	n.a.
Urologic problems during pregnancy	9.36	0.97–90.78	0.133	17.36	1.68–179.44	0.080	5.32	057–49.89	0.214
Oligohydramnios	n.a.	n.a.	n.a.	n.a.	n.a.	n.a.	n.a.	n.a.	n.a.
Gestational age at birth	1.06	0.23–4.96	1.000	n.a.	n.a.	n.a.	0.62	0.22–1.77	0.368
Vacuum delivery	0.39	0.11–1.30	0.111	1.06	0.23–4.77	1.000	0.63	0.26–1.56	0.317
Forceps delivery	2.77	0.58–13.27	0.202	2.25	0.26–19.35	0.401	2.49	0.69–9.03	0.157
Cesarean section	2.25	0.71–7.11	0.205	0.88	0.17–4.57	1.000	1.19	0.46–3.06	0.715
Newborn's gender	0.43	0.12–1.62	0.202	0.53	0.10–2.74	0.704	0.70	0.28–1.80	0.460
Birthweight	n.a.	n.a.	n.a.	n.a.	n.a.	n.a.	n.a.	n.a.	n.a.
Shoulder dystocia	9.36	0.97–90.78	0.133	n.a.	n.a.	n.a.	15.00	2.36–95.43	0.019
Neonatal sepsis	n.a.	n.a.	n.a.	n.a.	n.a.	n.a.	n.a.	n.a.	n.a.

Abbreviations: 1′ AS, first minute Apgar score; 5′ AS, fifth minute Apgar score; ANH, acute neonatal hypoxia; n.a., not applicable.

1. There was a statistically significant association between maternal multiparity and 1′AS ≤4, 5′AS ≤7 and ANH (OR: 4.45; 95% CI: 1.32–15.04; *p* = 0.022; OR: 5.47; 95% CI: 1.05–28.58; *p* = 0.037; and OR: 2.74; 95% CI: 1.11–6.77; *p* = 0.024, respectively)
2. There was a statistically significant association between shoulder dystocia at birth and ANH (OR: 15.00; 95% CI: 2.36–95.44; *p* = 0.019).

After the application of the logistic regression models, the conclusions were:

1. Belonging to group A did not affect the occurrence of 1′ AS ≤4, 5′ AS ≤7, or ANH when compared with group B (*p* = 0.185; *p* = 0.098; and *p* = 0.600, respectively);
2. There was a statistically significant impact of pH_ua _≤7.00 in the occurrence of ANH (OR: 6.71; 95% CI: 1.21–37.06; *p* = 0.029), but not in the occurrence of 1′ AS ≤4 or of 5′ AS ≤7 (*p* = 0.871; and *p* = 0.130, respectively);
3. Multiparous women had a statistically significant higher risk of delivering a newborn with 1′ AS ≤4 and ANH (OR: 5.38; 95% CI: 1.35–21.43; *p* = 0.017; and OR: 2.66; 95% CI: 1.03–6.89; *p* = 0.043, respectively);
4. Women who had urologic problems during pregnancy had a statistically significantly higher risk of delivering a newborn with 5′ AS ≤7 (OR: 15.17; 95% CI: 1.29–177.99; *p* = 0.030);
5. Shoulder dystocia represents a 15 times higher risk of ANH (OR: 14.82; 95% CI: 2.20–99.60; *p* = 0.006).

## Discussion

The pH_ua_ seems to be a reliable marker for acute peripartum problems and a good predictor of adverse neonatal outcomes, as has been widely documented in the literature.^1^ ^2^ ^4^ ^5^ ^6^ The results of the present study support these concepts. In fact, the newborns with pH_ua _≤7.00 had a 9.57 times higher risk of 1′ AS ≤4, a 10.83 times higher risk of 5′ AS ≤7, and a 7.6 times higher risk of ANH; and after the multivariate analysis, pH_ua _≤7.00 remained as an independent predictor of ANH, granting a 6.71 higher risk.

There is no consensus about the importance of BD_ua_ as an adverse outcome predictor.^3^ ^5^ ^6^ ^9^ In the present study, the risk of 1′AS ≤4 and of ANH was 27.4 and 7.44 higher for the newborns with BD_ua _≥12.00 mmol/L, respectively; and after excluding the confounding factors, BD_ua _≥12.00 mmol/L remained an independent predictor of 1′AS ≤4, increasing its risk almost 52 times.

Shoulder dystocia is a well-known risk factor of neonatal hypoxia. Therefore, it is not surprising that it significantly increases the risk of ANH. Performing an UCBGA seems warranted in these situations.

Although there was an association between pH_ua _≤7.00 and 1′AS ≤4, as well as between pH_ua _≤7.00 and 5′AS ≤7, belonging to group A did not affect the occurrence of 1′AS ≤4 or of 5′AS ≤7. This fact can be explained by the findings of Sabol et al,^1^ which concluded that newborns with a reassuring Apgar score have a residual risk of neonatal acidemia. They also concluded that, in this rare setting, the acidemic newborns have worse outcomes when compared with their non-acidemic counterparts.^1^ These findings support the introduction of universal UCBGA as a valuable neonatal screening test for neonatal hypoxia and its consequences.

Finally, the present study has found two other things that neither were expected nor clear in the previous literature. The newborns from multiparous women had a 5.38 and a 2.66 times higher risk of having 1′AS ≤4 and ANH, respectively, when compared with newborns from nulliparous women. One study of Mgaya et al^14^ also found an increased incidence of low Apgar scores in newborns from grand multiparas. These findings can indicate an increased risk of disturbances in the fetal oxygenation that would predispose fetuses to a lower oxygen reserve during labor, but more studies are necessary to understand and validate this association.

The only independent predictor for 5′AS ≤7 in the present study was urologic problems during pregnancy, which granted a 15.17 times higher risk of this adverse outcome. This finding is difficult to understand in the light of the current knowledge.

The present study has the limitations of a retrospective cohort: not all of the confounding variables can be controlled, and the data collected relies on accurate patient files, except for the UCBGA. In the 56 cases excluded due to limited data, there were 2 cases of shoulder dystocia and 23 cases of non-reassuring fetal CTG patterns, which may have caused a bias. Another limitation of the present study is that the UCBGA was not performed in all deliveries, but this seems to be shared in the majority of UBCGA studies. Collecting umbilical cord blood samples in emergency situations, like placental abruption or non-reassuring CTG patterns is difficult. In this study 66 out of the 148 UCBGA that were excluded because of incomplete, non-valid or only one-vessel results ocurred in such emergency settings, which lead to an inevitable bias. The small cohort size can justify why the only significant associations found were between pH_ua _≤7.00 and 1′AS ≤4; between 5′AS ≤7 and ANH; and between BD_ua _≥12.00 mmol/L and 1′AS ≤4 and ANH. Because only one newborn had HIE and there were no deaths, it became impossible to assess the relationship between these outcomes and neonatal pH_ua _≤7.00 or BD_ua _≥12.00mmol/L.

In the light of new evidence, the umbilical artery lactate level seems as a reliable outcome predictor of low Apgar score and of neurologic morbidity^6^ ^13^ that is directly measured from blood^3^. It is association with medium and long-term neonatal outcomes can be adressed in future.

Some recent studies argue that the threshold for adverse neonatal outcomes is pH_ua _≤7.10.^7^ ^8^ In the cohort of the present study, if the pH_ua_ threshold is changed from pH_ua _≤7.00 to pH_ua _≤7.10, the number of acidemic neonates increases from 17 to 79, so further studies can be designed to verify whether there is an association between pH_ua _≤7.10 and other neonatal outcomes besides 1′AS ≤4; 5′AS ≤7, and ANH.

Additional studies are necessary to understand if multiparity and urologic problems during pregnancy are true predictors of adverse neonatal outcome; and, if so, what are the responsible physiopathological mechanisms.

## Conclusion

The pH_ua_ and the BD_ua_ are predictors of adverse neonatal outcomes, and the UCBGA is a useful tool for screening newborns at risk. The meaning of low pH_ua_ and high BD_ua_ present in clinically healthy newborns with good Apgar scores remains to be determined, but the healthy acidemic newborns seem to have worse outcomes when compared with their non-acidemic counterparts. Therefore, universal UCBGA should be considered for all deliveries because it is an accurate screening test for neonatal hypoxia.
